# Evaluation of Immune Responses to Seasonal Influenza Vaccination in Healthy Volunteers in South Apulia, Italy: A Pilot Study

**DOI:** 10.4021/jocmr647w

**Published:** 2011-11-10

**Authors:** Quattrocchi Manuela, Lobreglio Gianbattista, Leuzzi Gianpiero, De Donno Antonella

**Affiliations:** a Laboratory of Hygiene, Department of Biological and Environmental Sciences and Technologies (DiSTeBA), University of Salento, Lecce, Italy; b Laboratory Medicine Unit, ”Cardinale Panico“ Hospital, Tricase, Lecce, Italy; c General Practitioner ASL LECCE, Lecce, Italy

## Abstract

**Background:**

The aim of our pilot study is to investigate different components of the immune response to influenza vaccination in a group of healthy volunteers. We evaluated the cellular immune response (CD4^+^ T lymphocytes) by flow cytometry. The humoral immune response was assessed by measuring the serum haemagglutination inhibition antibody response.

**Methods:**

Healthy adult donors (n = 18), were vaccinated with a commercially influenza virus vaccine (FLUARIX® GlaxoSmithKline S.p.a. Verona, Italy), peripheral blood was drawn the same day as influenza virus vaccination and one month later in order to enumerate the antigen-specific CD4^+^ T lymphocytes. Hemagglutination inhibition assay was performed to enumerate the titer of neutralizing antibodies. Samples of nasal-pharyngeal secretions were taken by swabbing, from ILI (Influenza like Illness) subjects among the studied group, in order to verify influenza infections and eventually identify viruses using Real Time PCR.

**Results and Conclusions:**

Parenteral influenza vaccination results in significant increase in the CD4^+^ Th cell population after vaccination. The number of pre-vaccination CD4^+^ T cells was 0.018 [the results are presented as number of percent fluorescent cells per 10 000 lymphocytes (fixed cells)], while there was a significantly higher number of CD4^+^ Th cells one month after vaccination (statistical significance was set at the level of α = 0.01). Twenty-two percent of patients demonstrated protective antibody levels to influenza A H1/N1 serotype. None was diagnosed with influenza type A or B.

**Keywords:**

T cells; Flow cytometry; Influenza virus; Influenza vaccine

## Introduction

Influenza virus is a common respiratory pathogen that causes substantial morbidity, hospitalization and mortality worldwide every year especially in the case of infants, the elderly and immuno-compromised patients [1,2]. The incidence of the illness depends on the immunity acquired by previous exposure (infection or vaccination) to the circulating strain. Each year, 10% to 20% of the population is infected. In clusters (schools, healthcare facilities, etc.), incidence can reach 40 - 50%. At the present, the infection mainly affects the young population ≤ 14 years and adults in the 15 - 64 years old age group, while influenza-related complications occur most frequently in the elderly [3,6]. Resistance to influenza virus infection and disease is mediated by an intricate pattern of innate and acquired immunity with both the local (mucosal) and the systemic arms involving a variety of immuncompetent cells including B cells (humoral immunity), T cells (cellular immunity), antigen presenting cells and matrix cells. Antibodies secreted locally, particularly secretory IgA (S-IgA), in the upper respiratory tract are a major factor in resistance to natural infection [7], whereas serum IgG plays an important role in protection of the lower respiratory tract. Cell-mediated immunity is important in recovery from influenza infection and may also prevent influenza-associated complications, but it does not seem to contribute significantly to prevention of infection. Influenza infection induces a strong CD4^+^ T-helper (Th) response, which plays an important role in stimulating antibody production against the virus [8].

Influenza causes a broad spectrum of illness in humans, ranging from symptomless infection to fulminant primary viral or secondary bacterial pneumonia.

Despite the increasing availability of antivirals, vaccination is still the most cost-effective prevention alternative.

Vaccinaton is the principal way of preventing influenza and its complications, but it is less effective in immunocompromised patients, compared with healthy individuals [9,11,12].

The inactivated influenza virus vaccine, used since 1945, has been generally well tolerated and has been reported to induce substantial levels of protection, in the range of 70% to 90% when the vaccine and circulating wild-type strains are antigenically similar [13].

At present, the trivalent inactivated influenza virus vaccine (TIV), produced by several manufacturers, is licensed worldwide and recommended for many populations, including children 6 months to 5 years, adults over the age of 50, people with a variety of chronic illnesses, and health care workers.

Improvements could be made in the current vaccines effectiveness, duration of response, ease of administration and compliance. Thus, it is important to have a detailed knowledge of the protective immunity and immune processes occurring in the upper respiratory tract in man to allow development of new vaccines and vaccination strategies [14].

The present preliminary study was carried out to investigate different components of the immune response to influenza vaccination in a group of healthy volunteers. We evaluated the cellular immune response (CD4^+^ T lymphocytes) by flow cytometry. The humoral immune response was assessed by measuring the serum haemagglutination inhibition antibody response.

## Patients and Methods

### Study population

Eighteen healthy volunteers [7 men and 11 women, the median age 36.8 (27-55) years] were recruited by General Practitioners. Ten controls, unvaccinated healthy adults (median age, 35 years; range, 27 - 45 years) tested before and 4 weeks after influenza vaccination were included in the study.

### Influenza virus vaccination

Between October and November 2007 all the people studied were vaccinated with a commercially available inactivated influenza virus vaccine (FLUARIX® GlaxoSmithKline S.p.a. Verona, Italy) following current guidelines for influenza vaccination. This trivalent vaccine included the formaldehyde-inactivated influenza virus strains A/Solomon Islands/03/2006 (H1N1), A/Wisconsin/67/2005 (H3N2) and B/Malaysia/2506/2004; a dose of 0.5 mL contains 15µg of each virus strain.

### Analysis of lymphocyte populations by flow cytometry

Peripheral blood was drawn in EDTA-containing tubes the same day as influenza virus vaccination and one month later. Phenotypic detection of T cells was performed by three-color flow cytometry; to stain cell surface molecules, 100 μL of anti-coagulated blood were incubated for 2 h at room temperature (RT) in the dark with R-PE-labeled DRB1*0101(PKYVKQNTLKLAT)-restricted MHC Class II UltimersTM containing peptide derived from Influenza hemaglutinin (DR4/HA 306-318) that allowed the enumeration of antigen-specific CD4^+^ T lymphocytes (Proimmune Ltd., Oxford, UK) and CD4 FICT monoclonal antibody.

The assay was performed according to the manufacturers’ specifications. First, the erythrocytes were lysed with Lysing buffer 1X (BD Pharm Lyse^TM^) and the leukocytes fixed by incubating for 10 minutes at RT with 300 μL of Fix solution (1% fetal calf serum, 2.5% formaldehyde in PBS).

For the analysis of T cell subpopulation, multiparametric flow cytometry was performed by using a FACSCanto flow cytometer (Becton Dickinson, California). A total of 10 000 live events were acquired, gated on small viable lymphocytes and analyzed with BDFacsDIVA software (version 4.1.2) (Becton Dickinson, California). The instrument was routinely calibrated according to the manufacturer’s instructions.

The results are presented as number of percent fluorescent cells per 10 000 lymphocytes (fixed cells).

### Serum antibody titers

The hemagglutination inhibition assay was performed on serum samples using a single stock source for each of the HA antigens representing the strains of virus contained in the vaccine [11]. The antibodies titers were determined before and 4 weeks after vaccination. Immunity to influenza was defined as a HI titer more than or equal to 40.

### Controls

Whole blood from unvaccinated patients were treated in the same manner as the samples from vaccinated patients.

### Analysis of influenza viruses by Real Time PCR

Between December 2007 and March 2008, samples of nasal-pharyngeal secretions were taken, by swabbing, from ILI (Influenza like Illness) subjects among the studied group, in order to verify influenza infections and eventually identify viruses using Real Time PCR.

During 2007-2008 influenza season a real-time RT-PCR reaction was performed on the studied samples; both the reverse transcription and PCR steps were reacted in the same tube; primers and probes for influenza viruses A and B were based on genomic regions highly conserved in various subtypes and genotypes of influenza virus A matrix protein gene) and influenza virus B (haemagglutinin gene segment) (Fast set InfA/InfB-Arrow Diagnostics S.r.l., Genova, Italy) [15].

### Statistical analysis

Pre-post-vaccine comparisons were done using ANOVA test. Statistical significance was set at the level of α = 0.01.

## Results

### Analysis of lymphocyte populations by flow cytometry

CD4^+^ Th cells are pivotal cells in the immune system, secreting cytokines to control and regulate the immune system. Parenteral influenza vaccination results in significant increase in the antigen-specific CD4^+^ Th cell population after vaccination.

**Figure 1:**
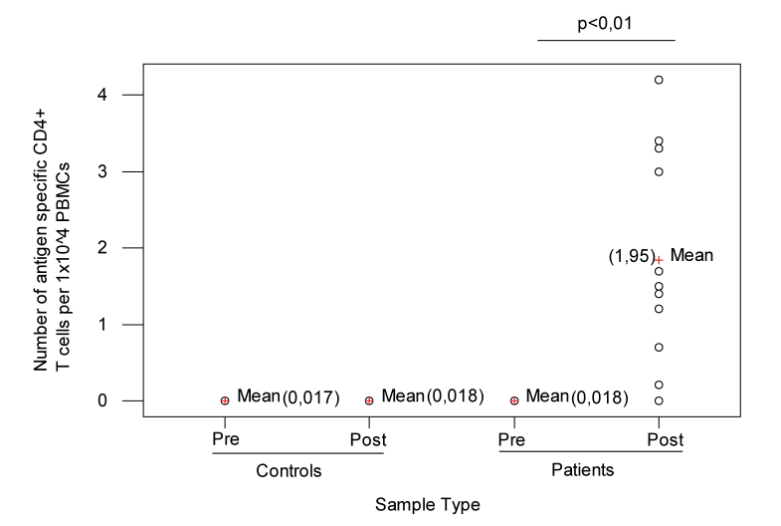
Controls and patients before and after vaccination.

The number of antigen-specific CD4^+^ T cells lymphocytes in the peripheral blood was determined by flow cytometry. As shown in Figure 1 number of pre-vaccination CD4^+^ T cells was 0.018 (range, 0 - 0.03%) (Median = 0.02%, StDev = 0.01). This value is consistent with the hypothesis that most if not all of the donors had been exposed to influenza during their lifetime. There was a significantly higher number of antigen-specific CD4^+^ Th cells one month after vaccination; the mean of percentage of number of post-vaccination CD4^+^ T cells was 1.95 (range, 0 - 4.2%) (Median = 1.5%, StDev = 1.26) and ANOVA test presents a P = 0 (P < 0.01); when T0 and T30 data were compared, no significant differences in the peripheral blood count of antigen-specific CD4^+^ T cells lymphocytes in control subjects were detected.

### Serology

Serum antibody titers were measured pre- and postvaccination. No patient had a protective antibody level against influenza-A before vaccination. One patient had prevaccination influenza-B-specific IgG level more than or equal to 20. Four weeks after the immunization 4 of 18 patients (22%) demonstrated protective antibody levels to influenza A H1/N1 serotype, whereas none of the patients was immune to H3/N2 serotype or influenza B (Table 1).

**Table 1:** Serum Antibody Titers Measured Pre- and Postvaccination

****	**Serum antibody titers before vaccination**	**Serum antibody titers after vaccination**
Influenza AH1/N1 IgG	-	4 (22%)
Influenza AH3/N2 IgG	-	-
Influenza B IgG	1 (5%)	-

### Analysis of influenza viruses by Real Time PCR

Of the 18 healthy donors and of the 10 controls, none was diagnosed with influenza type A or B.

## Discussion

Influenza viruses are unique in their capacity to cause recurrent illnesses, yearly epidemics and more extended pandemics that spread quickly and can have an effect on all population groups. Currently, there are two options to control influenza-vaccination and treatment with antiviral drugs. During the past decade there have been new developments in improving the efficacy of influenza vaccination, including the construction of live attenuated, recombinant or nucleic-acid vaccines [2]. The effectiveness of protective antibodies specific for HA for both treatment and prophylaxis of influenza A infection has been shown in animals models [16-18], but use of animal-derived antibodies in humans is limited because of severe anaphylactic reactions [19].

Thus, vaccination with inactivated vaccine remains the main strategy for influenza prevention but it might fall short in immunocompromised individuals. Therefore, monitoring the state of the immune system is a vital element in our understanding of disease progression and pathology.

CD4 cells are an important component of the anti-viral response to local and systemic infections.

More recent studies have determined that CD4 cells were necessary for long lasting, effective CD8 memory.

CD4 effector cells can also promote survival to a lethal dose of influenza infection and may contribute to immune-mediated pathology; CD4 effector T cells and memory contribute to immunity to influenza via multiple mechanism [20].

Recent studies have recognized intrinsic limitations of the serological methods currently used as a sole measure to evaluate influenza virus vaccine efficacy, and it has been suggested that evaluation of the cellular immune response could provide additional information to enable better estimation of protection against the disease [21,22]. We monitored the precursor frequency of the proliferating CD4^+^ T cells in response to specific antigen stimulations by using the flow dye dilution assay in 18 healthy donors, 0-30 days post vaccination with inactivated influenza virus vaccine.

This test, compared to traditional methods for assaying antigen-specific proliferation, offers the additional ability to evaluate the phenotype of the responding cells and is able to determine their rate of proliferation.

The sensitivity of the cytometric assay is likely the result of a combination of factors, including (a) the high sensitivity of fluorescence detection by modern flow cytometers; (b) the highly efficient capture of produced cytokine within the cytoplasm of the sectretion-inhibited responding cell; (c) the independence of culture conditions and the single cell signal detection strategy, allowing these conditions to be set up with optimization of response as the only concern; and (d) the relatively short stimulation period (4 h) [23].

All of the adults and elderly enrolled in our pilot study demonstrated that vaccination against seasonal influenza induce cellular immunity against influenza viruses. We do not found significant differences in the number of CD4^+^ T cells lymphocytes responses elicited after immunization between subjects among the studied group.

Among control subjects, the frequency of proliferating antigen-specific CD4^+^ T cells was essentially the same at T0 and T30; in contrast, among healthy donors, a statistically significant increase in the frequency of proliferating T cells was already detected at day 30 post-vaccination.

Based on this body evidence, we conclude that MHC Class II tetramers can be used to reliably detect CD4^+^ T cells specific for prevalent pathogens in normal donors. Regards serology, in our study with only 22% of patients developing a titer to H1N1 generally accepted as being protective, although no patient developed protective titers to either H3N2 or influenza B. The low response could be due to the fact that these are previously unvaccinated, and therefore would need a second dose of vaccine, on the contrary, as evidenced by our data, a single dose of vaccine would be effective anyway to make the priming of CD4^+^ T lymphocytes in the majority of vaccine subjects.

Cell-mediated immunity may also play a role in competition among influenza strains. Althoug T lymphocytes do not confer clinically significant protection against infection in humans, they can mediate cross-reactive and heterotypic protection in responce to conserved viral proteins in mouse models, and reduced viral shedding has been seen in the absence of antibodies against HA and NA. Cytotoxic T lymphocytes that are generated by seasonal influenza viruses against conserved epitopes might provide heterotypic immune responses that could dampen transmission, even in the absence of measurable antibody protection [24].

In conclusion, our study shows that cell-mediated immunity is important in recovery from influenza infection and may also prevent influenza-associated complications. Vaccination remains a priority, improved vaccines are needed, especially for vulnerable patients who are at increased risk of hospitalisation such as infants, the immunosuppressed and the elderly.

However, to establish if cellular immune response is able to provide an adequate level of protection against influenza would require further studies involving a larger number of subjects and for a longer time.
